# Supplementary figures and images for: Targeting an anchored phosphatase-deacetylase unit restores renal ciliary homeostasis
Source: eLife. 2021 Jul 12;10:e67828. doi: 10.7554/eLife.67828 (PMC8291974; doi:10.7554/eLife.67828)

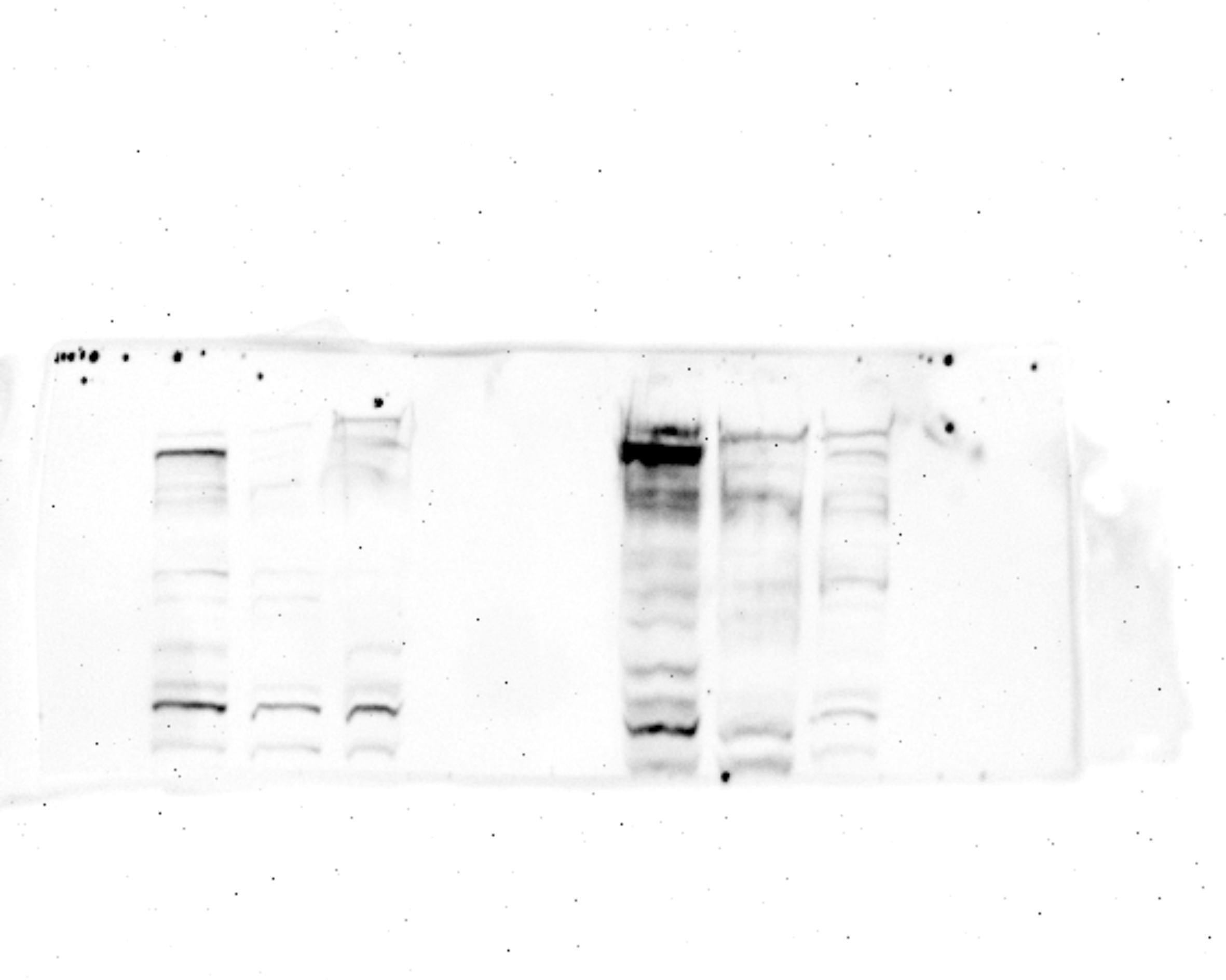

Supplement: Source data 1. [file elife-67828-data1.zip › original Figure 1K (2).png]

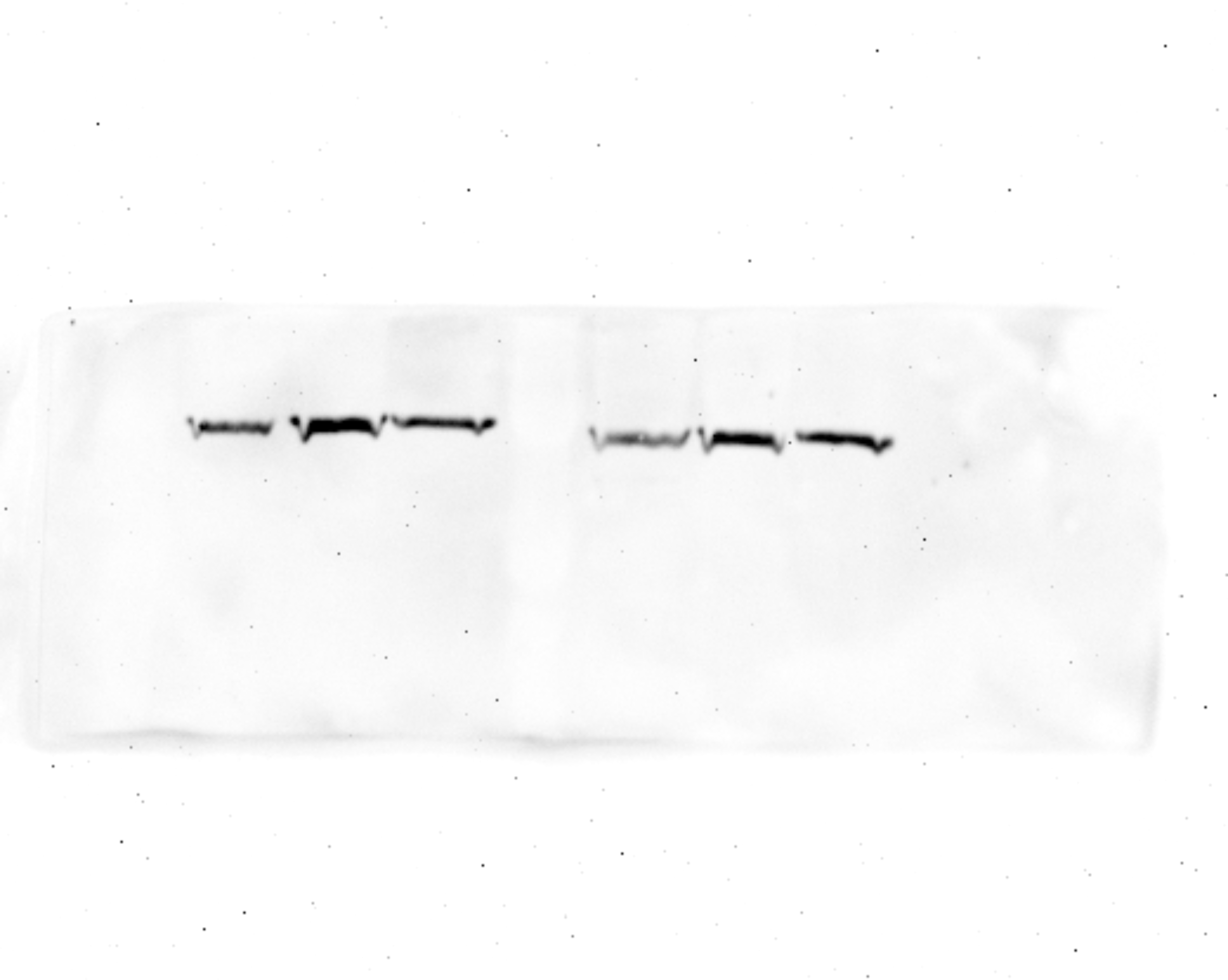

Supplement: Source data 1. [file elife-67828-data1.zip › original Figure 1K.png]

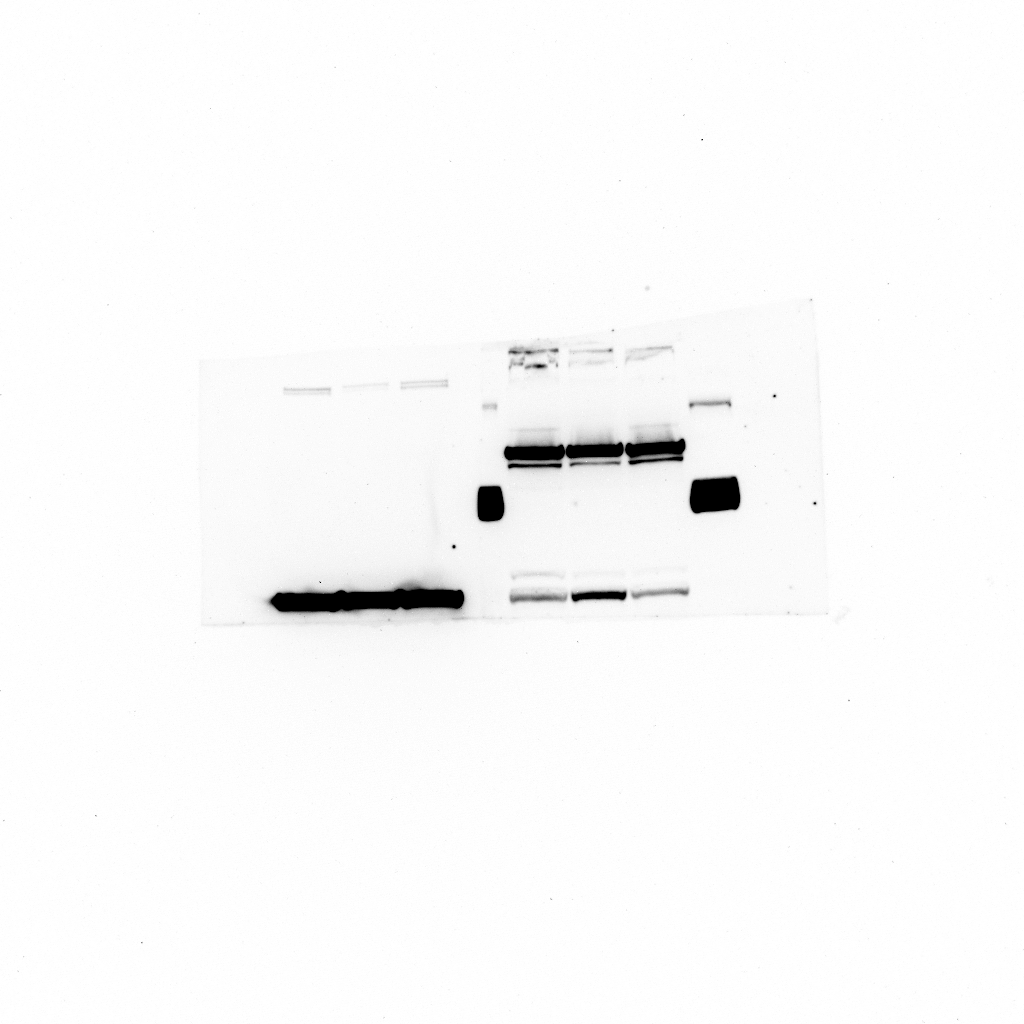

Supplement: Source data 1. [file elife-67828-data1.zip › original Figure 2C (2).tif]

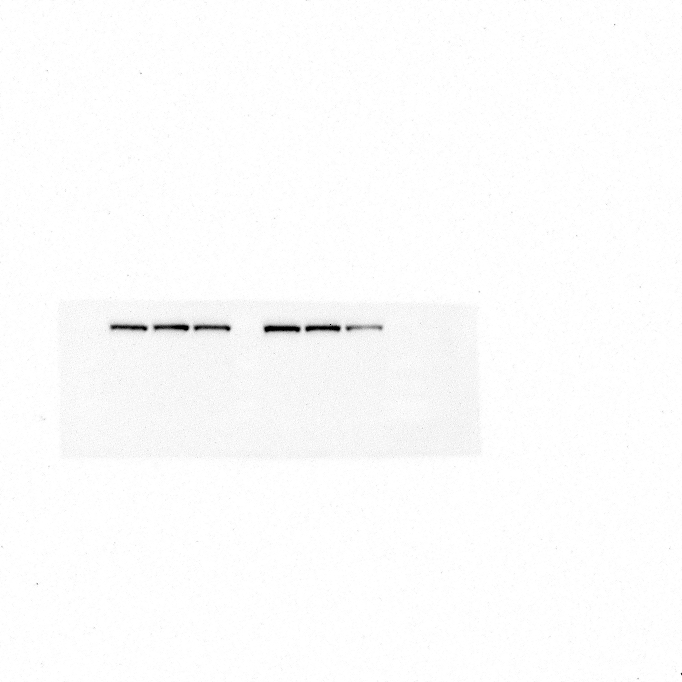

Supplement: Source data 1. [file elife-67828-data1.zip › original Figure 2C.tif]

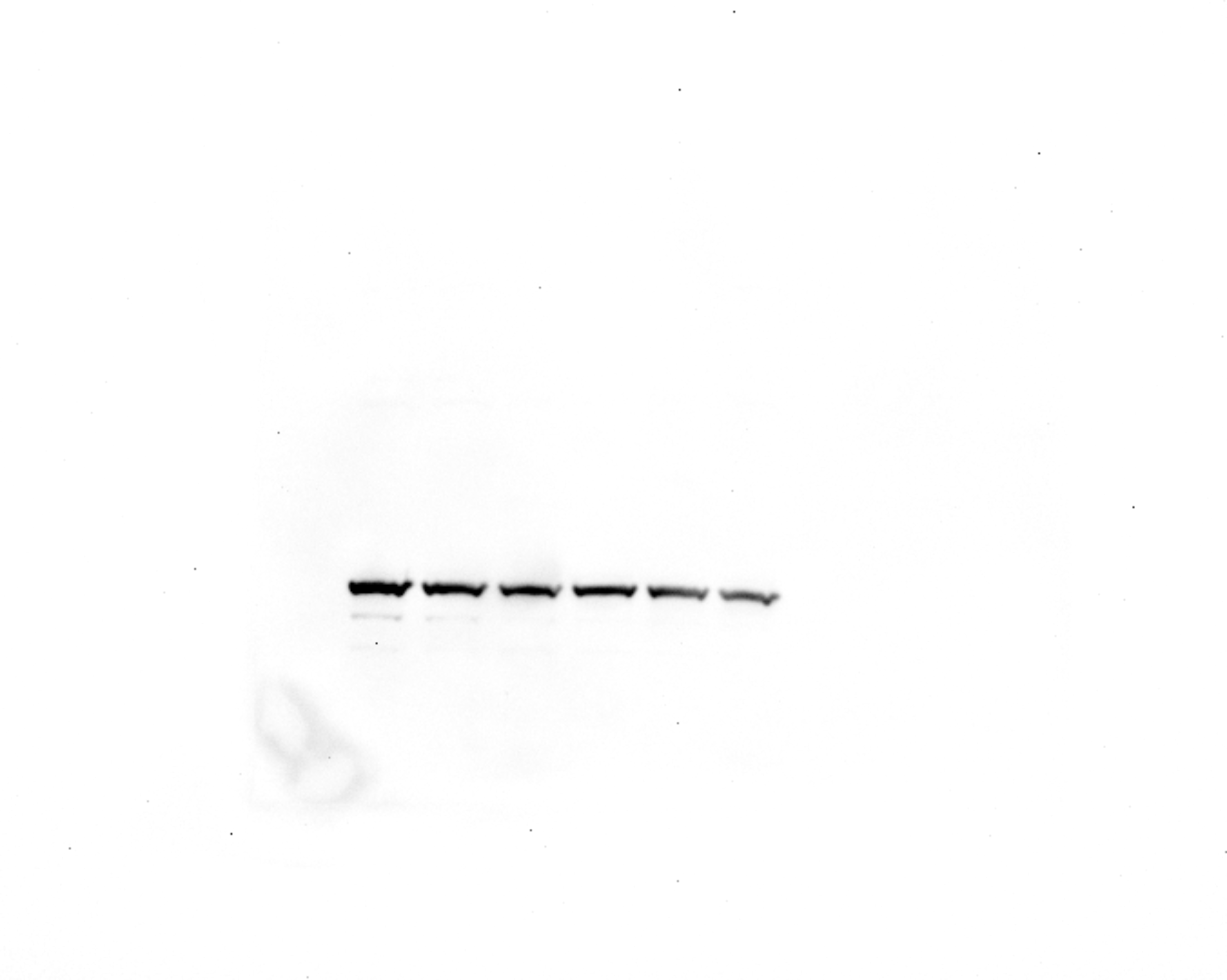

Supplement: Source data 1. [file elife-67828-data1.zip › original Figure 2I (2).png]

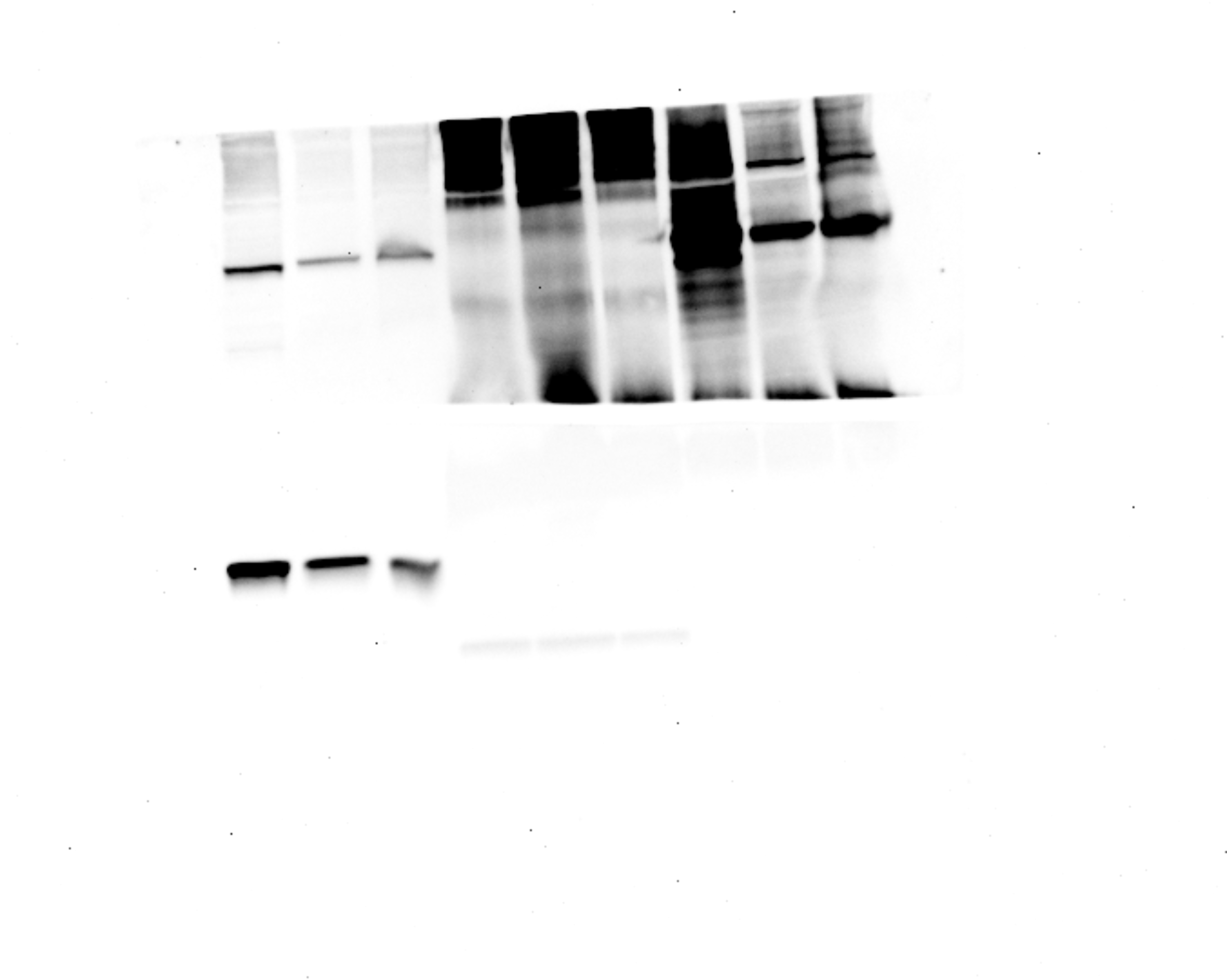

Supplement: Source data 1. [file elife-67828-data1.zip › original Figure 2I.png]

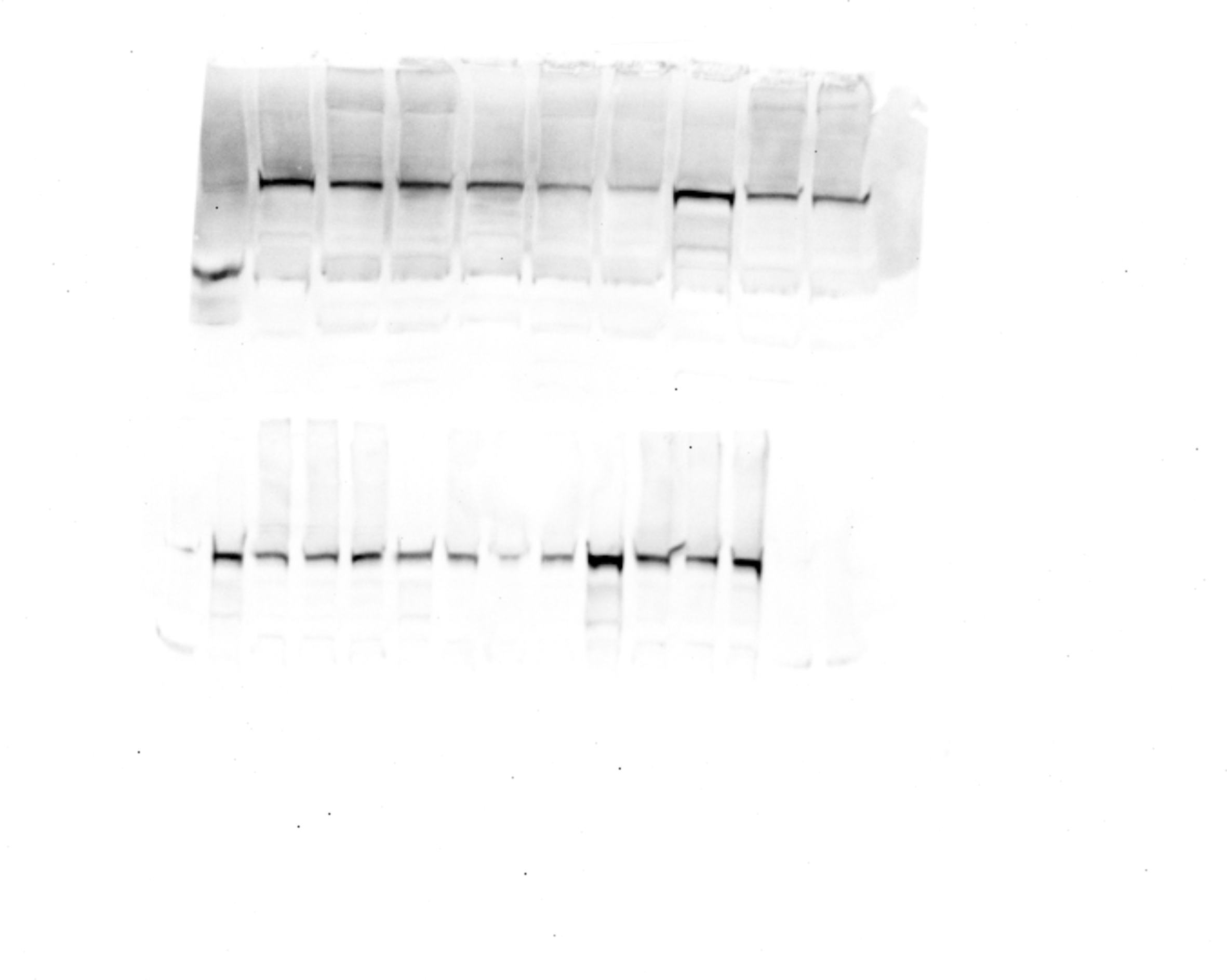

Supplement: Source data 1. [file elife-67828-data1.zip › original Figure 2P (2).png]

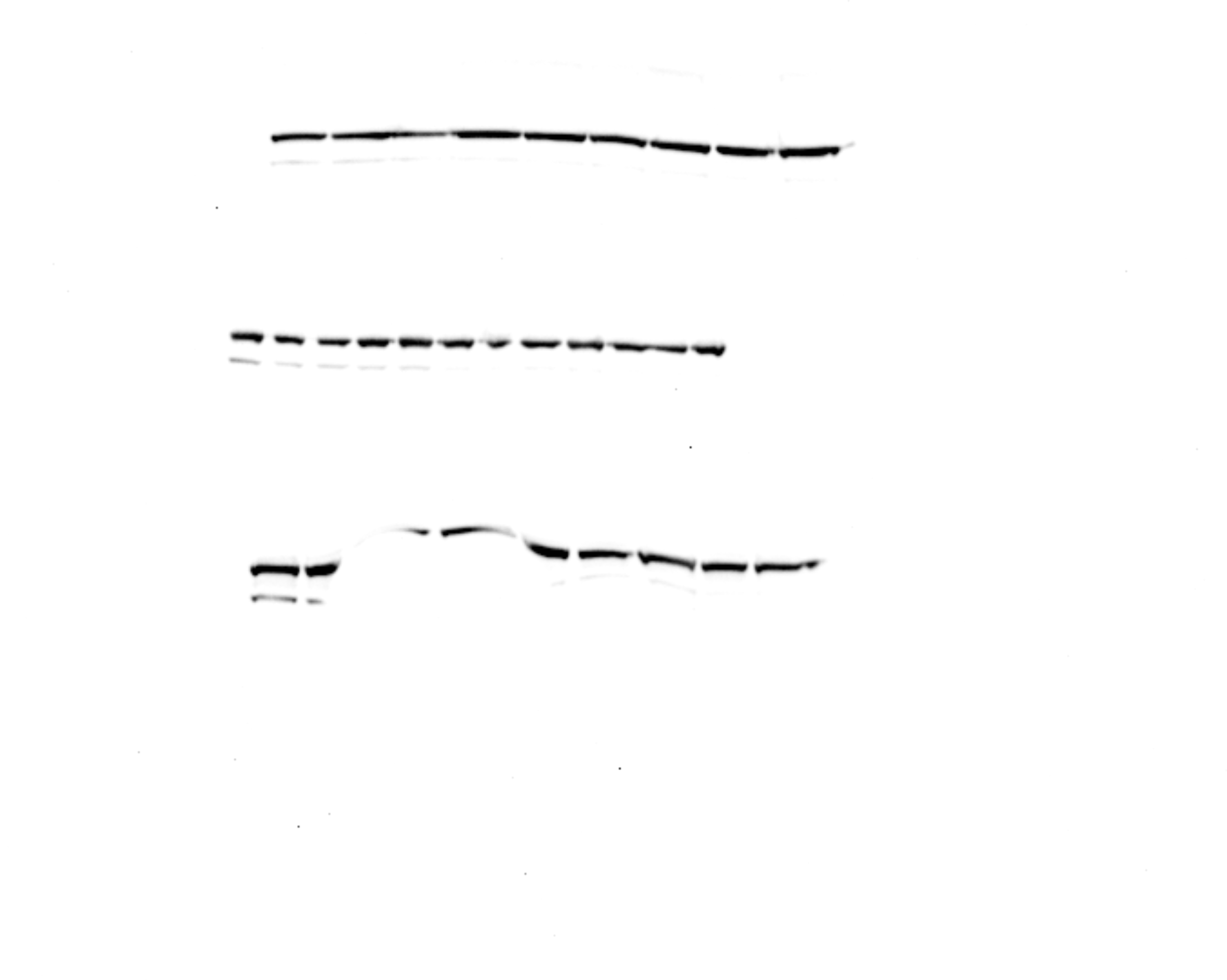

Supplement: Source data 1. [file elife-67828-data1.zip › original Figure 2P.png]

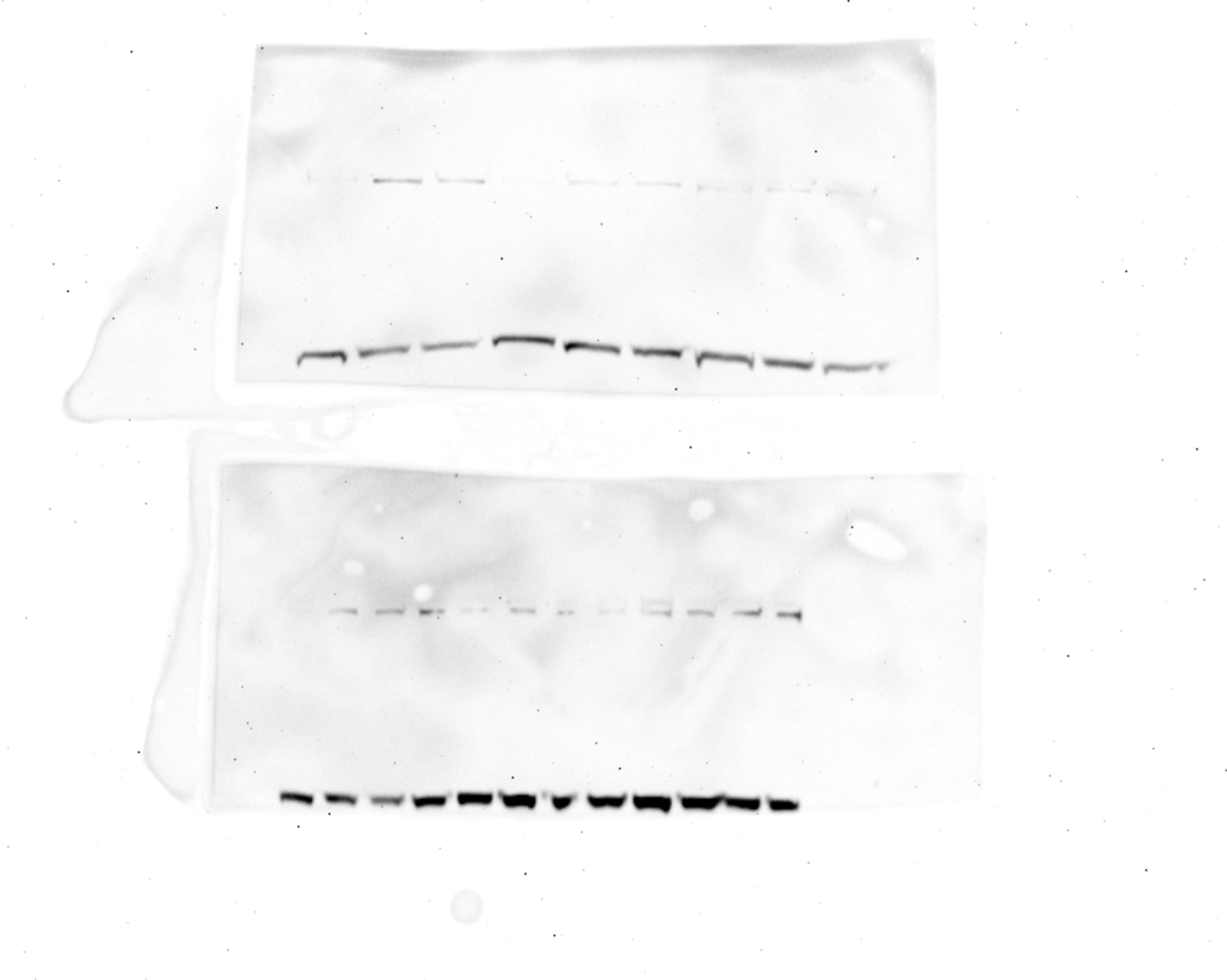

Supplement: Source data 1. [file elife-67828-data1.zip › original Figure 2P.tif]

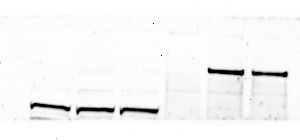

Supplement: Source data 1. [file elife-67828-data1.zip › original Figure 3- figure supplement 1C (2).tif]

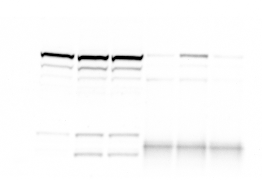

Supplement: Source data 1. [file elife-67828-data1.zip › original Figure 3- figure supplement 1C.tif]

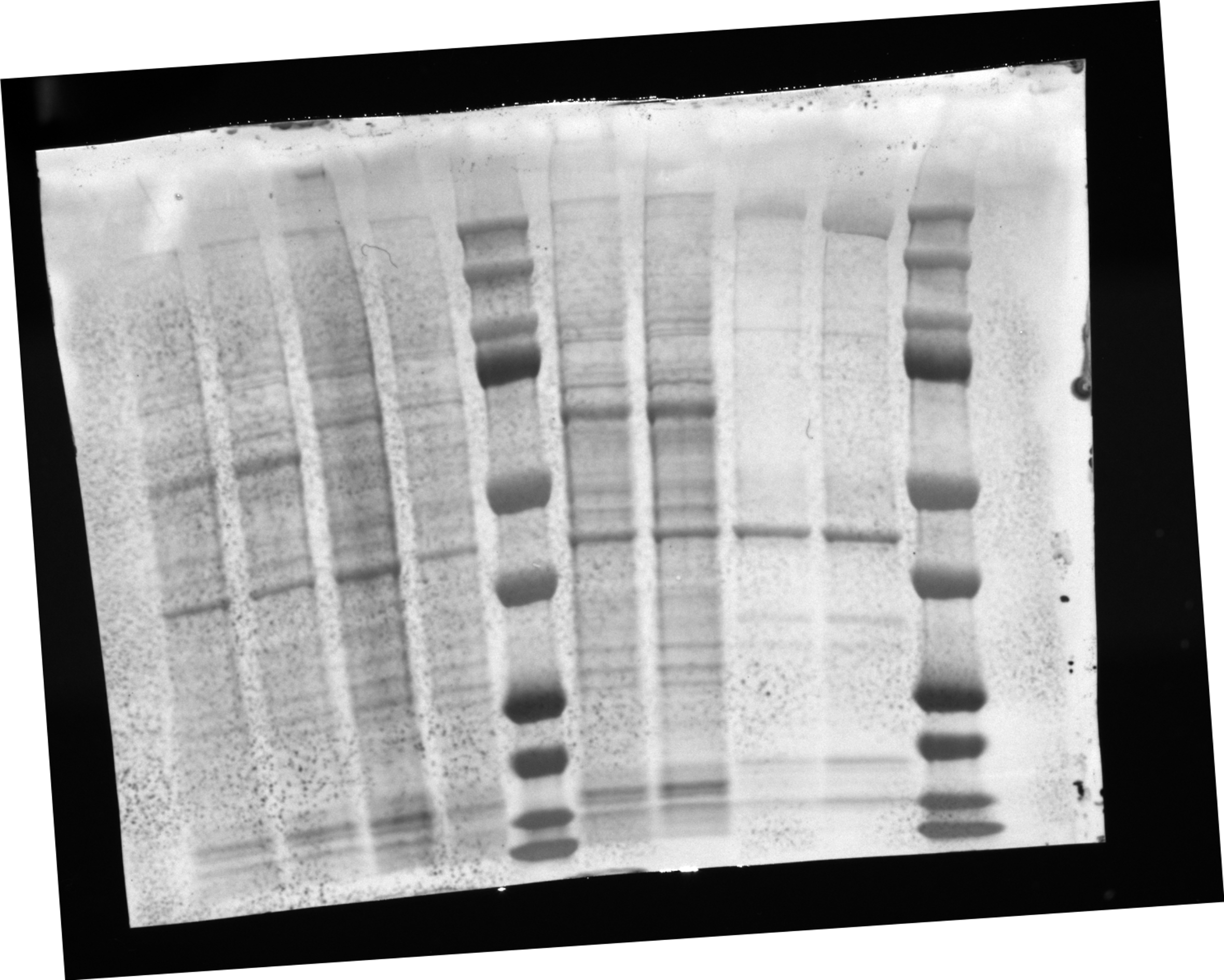

Supplement: Source data 1. [file elife-67828-data1.zip › original Figure 3- figure supplement 2A.png]

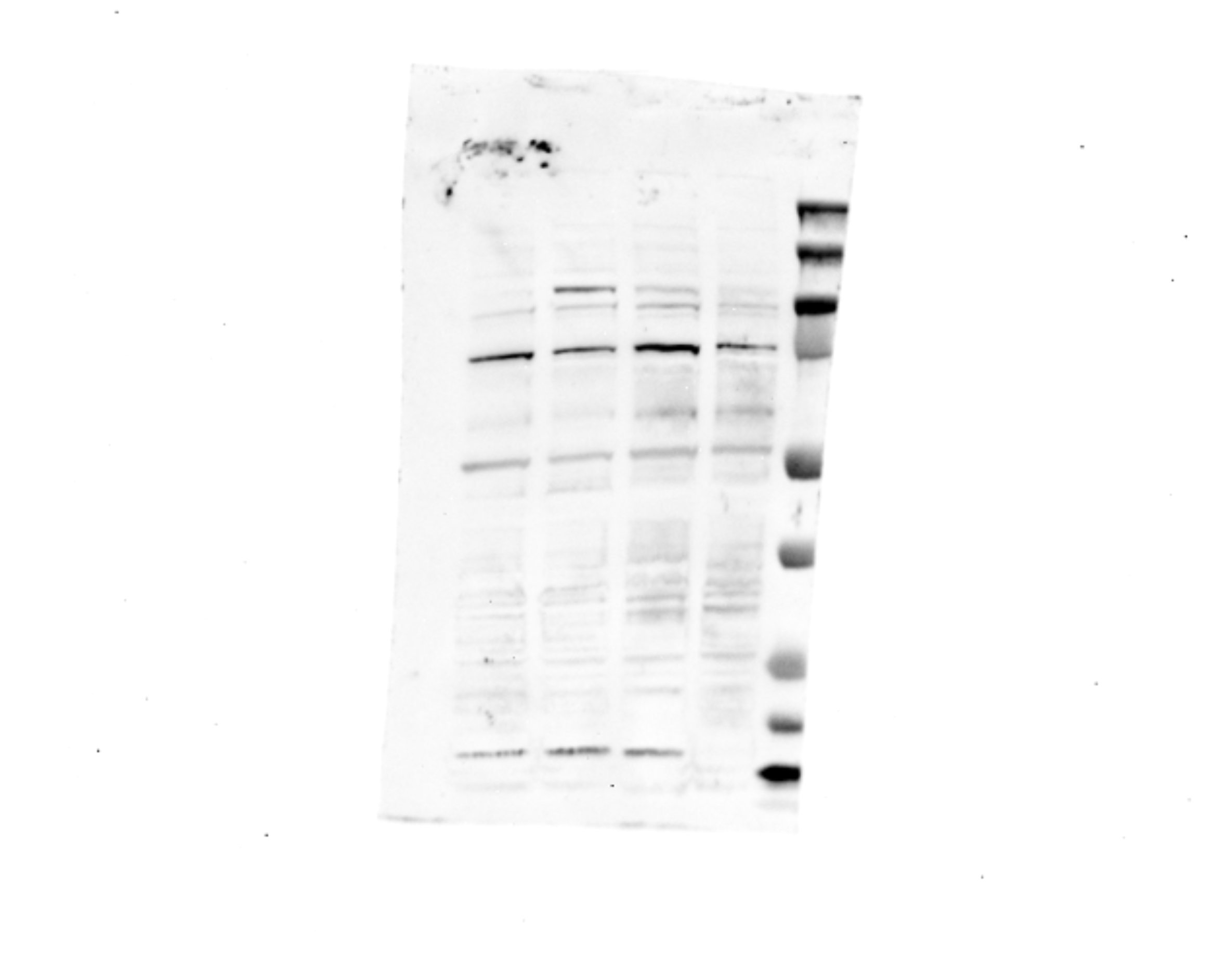

Supplement: Source data 1. [file elife-67828-data1.zip › original Figure 3- figure supplement 2A.tif]

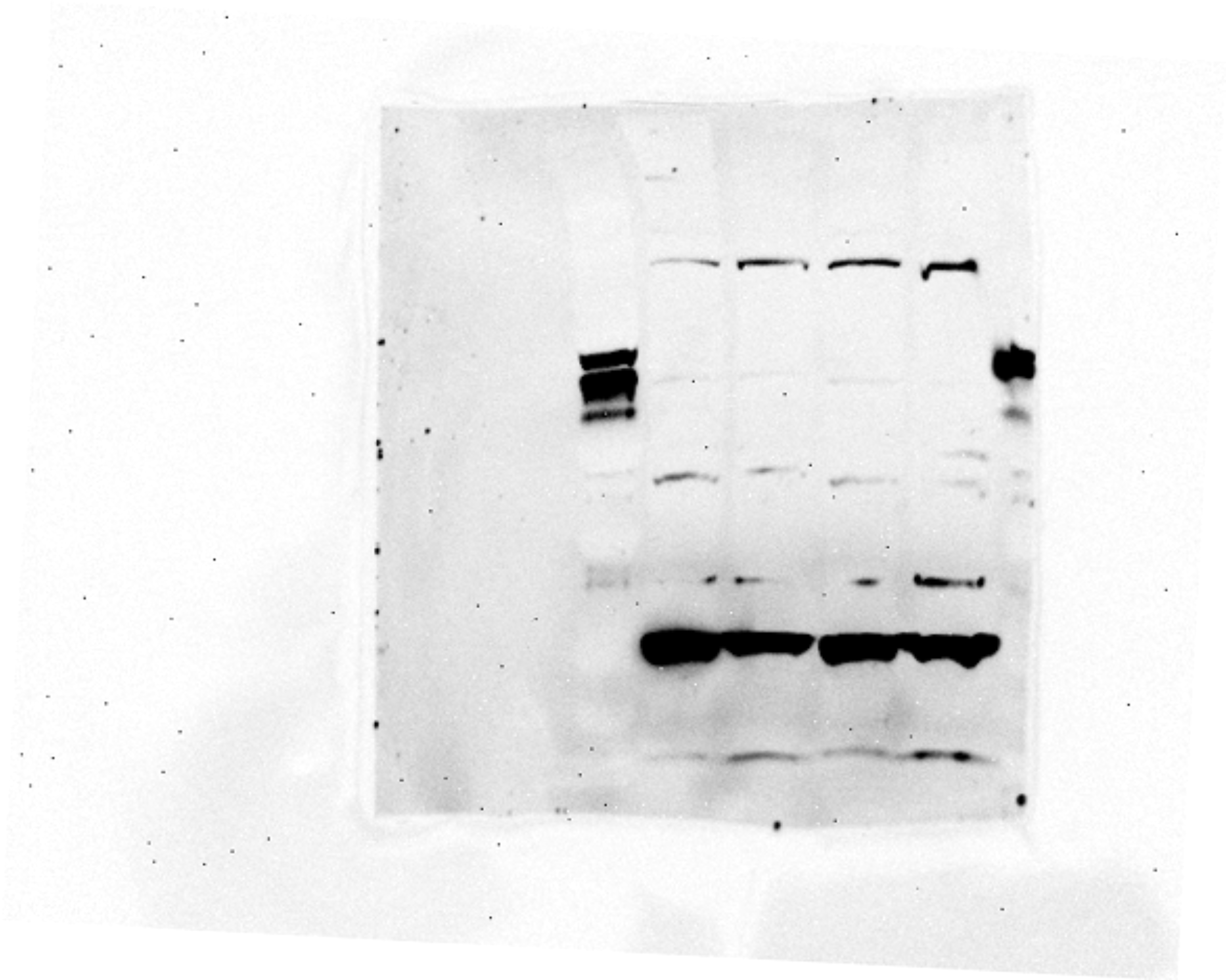

Supplement: Source data 1. [file elife-67828-data1.zip › original Figure 3- figure supplement 2B (2).png]

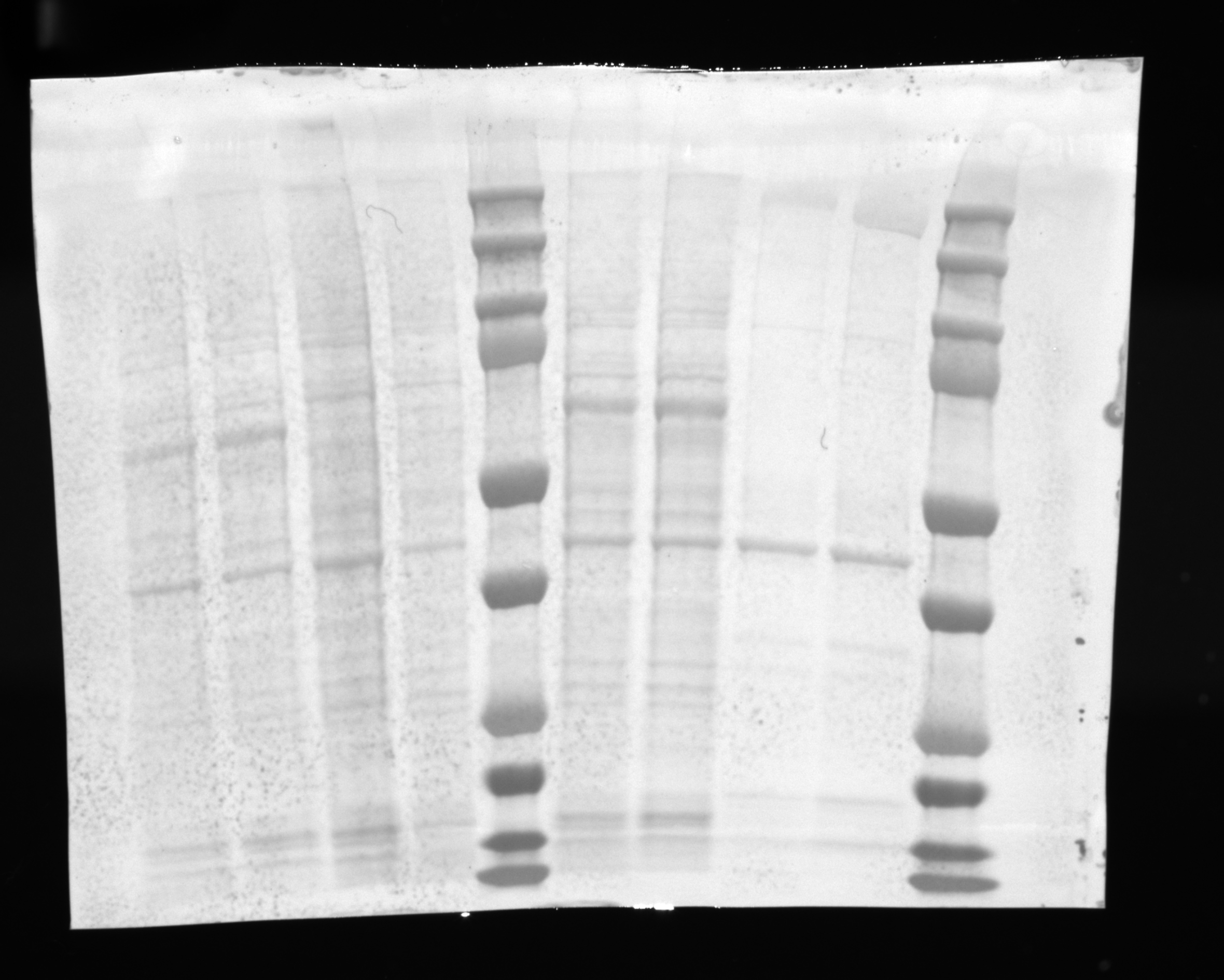

Supplement: Source data 1. [file elife-67828-data1.zip › original Figure 3- figure supplement 2B.png]

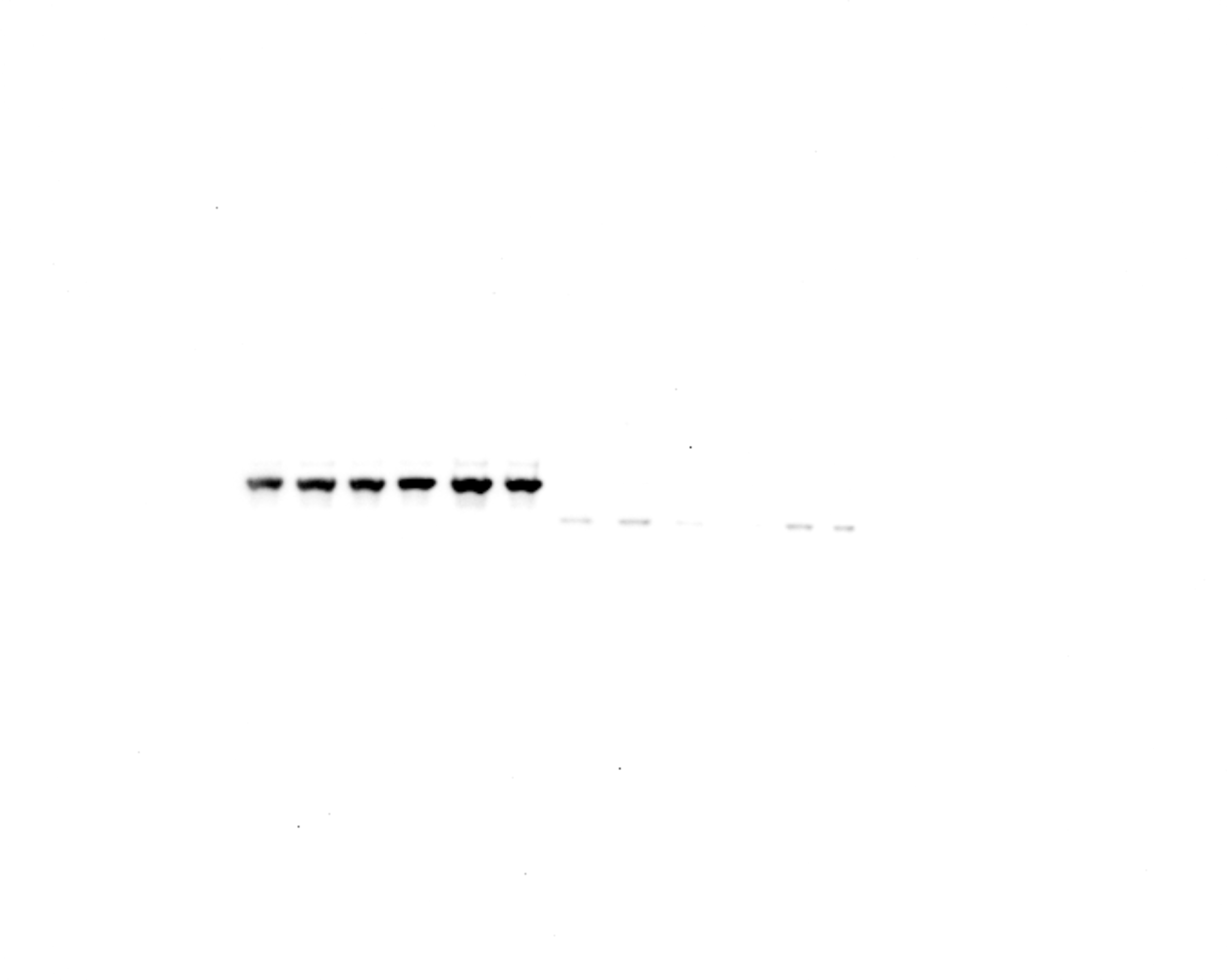

Supplement: Source data 1. [file elife-67828-data1.zip › original Figure 3C (2).tif]

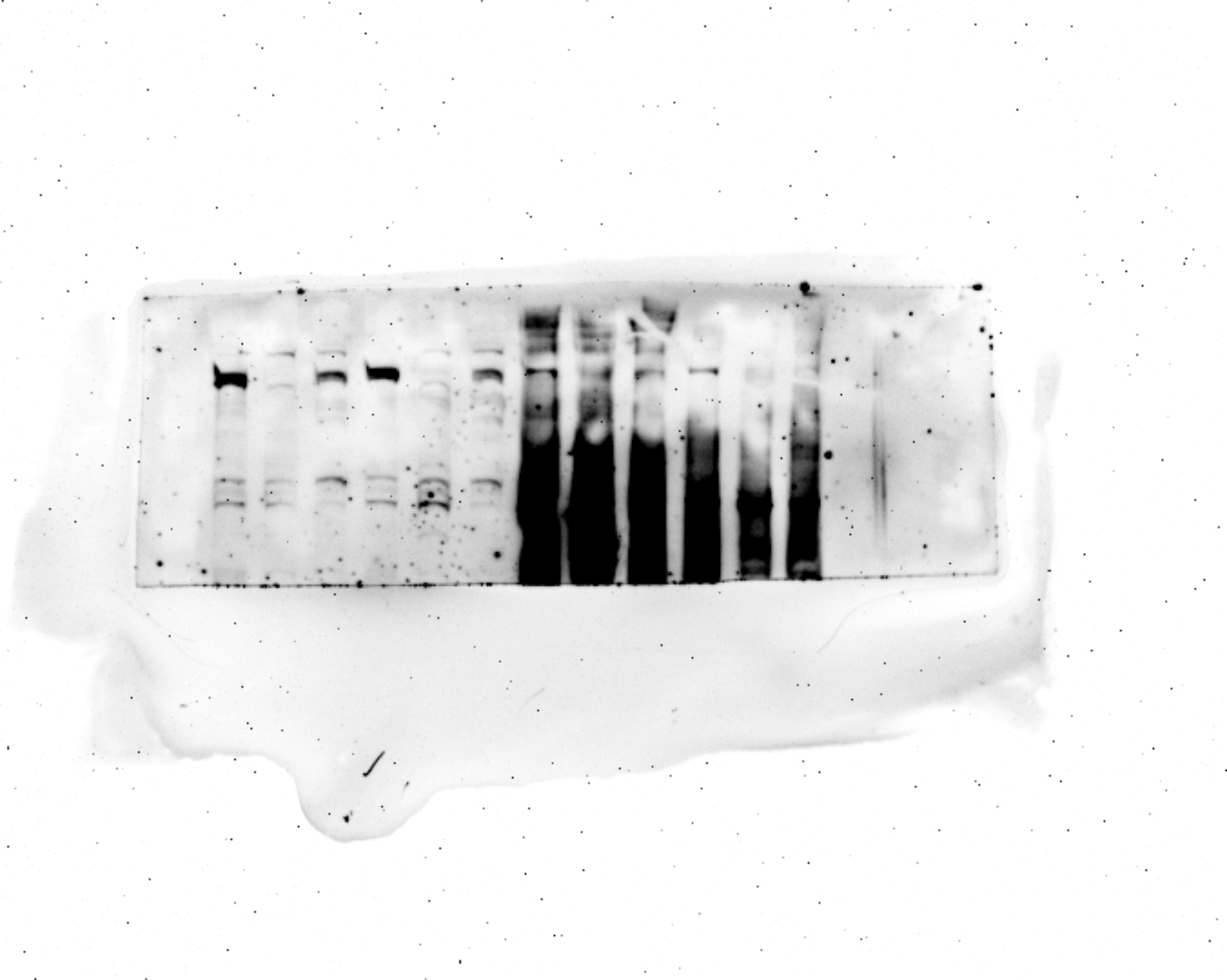

Supplement: Source data 1. [file elife-67828-data1.zip › original Figure 3C.tif]

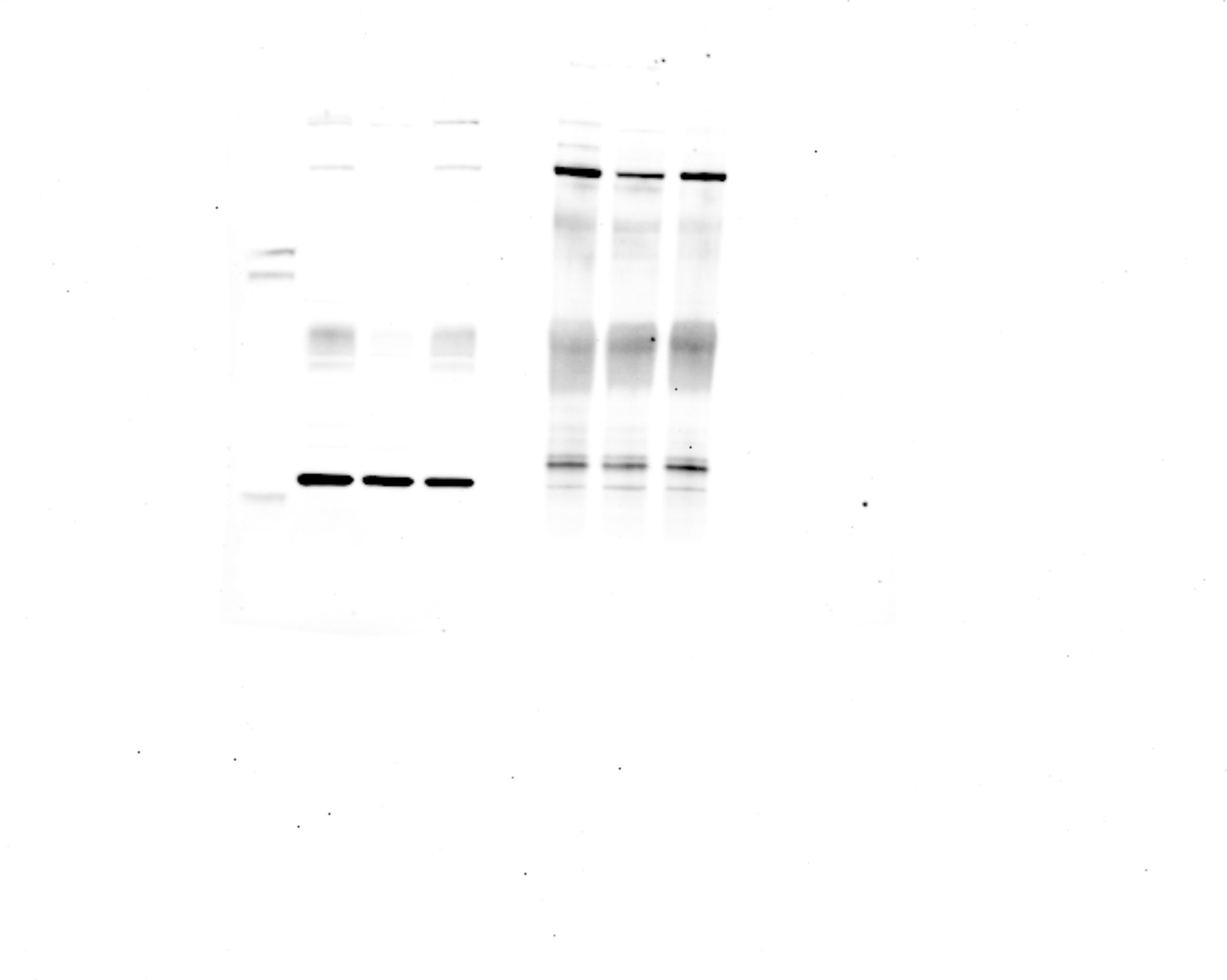

Supplement: Source data 1. [file elife-67828-data1.zip › original Figure 3D (2).tif]

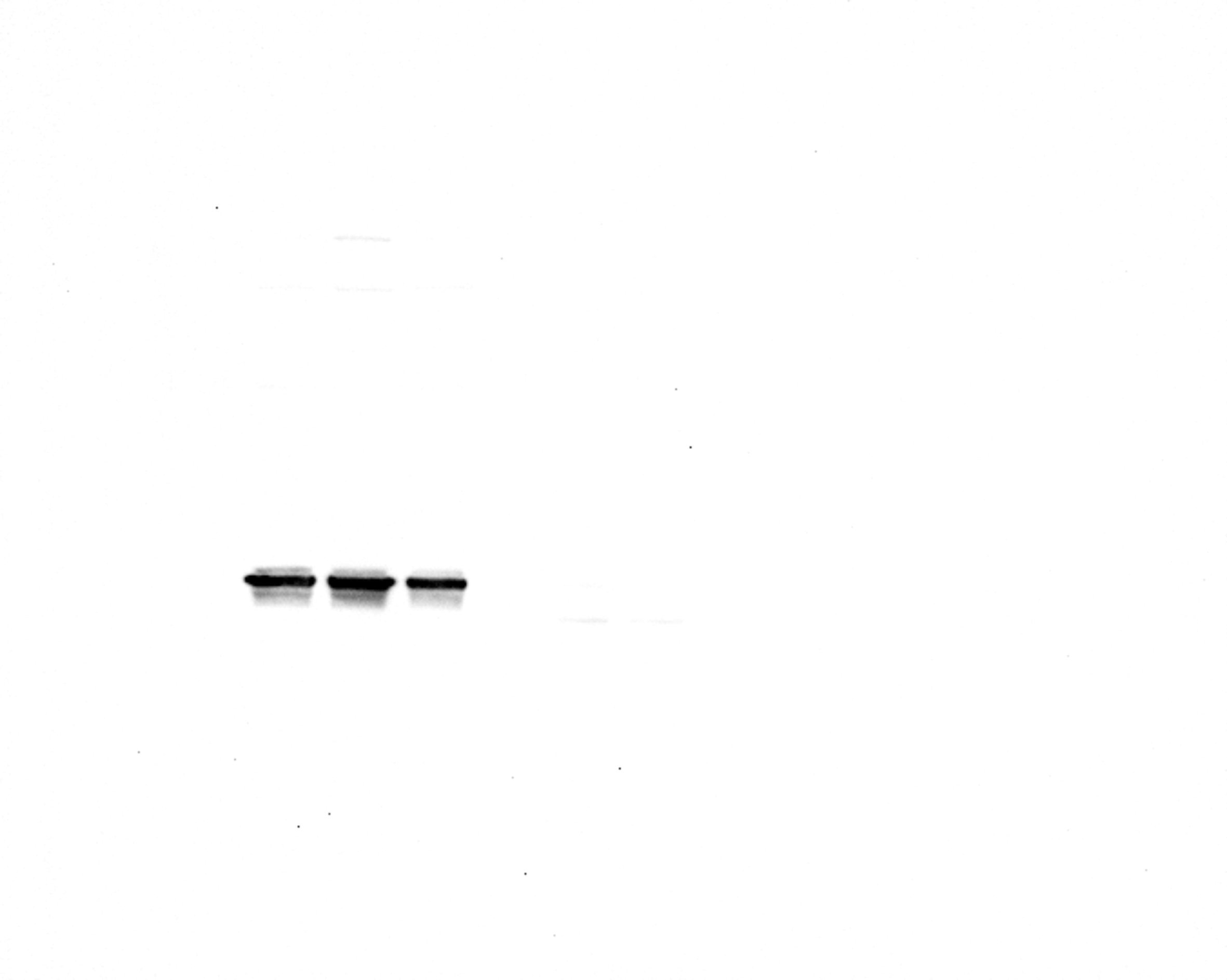

Supplement: Source data 1. [file elife-67828-data1.zip › original Figure 3D (3).tif]

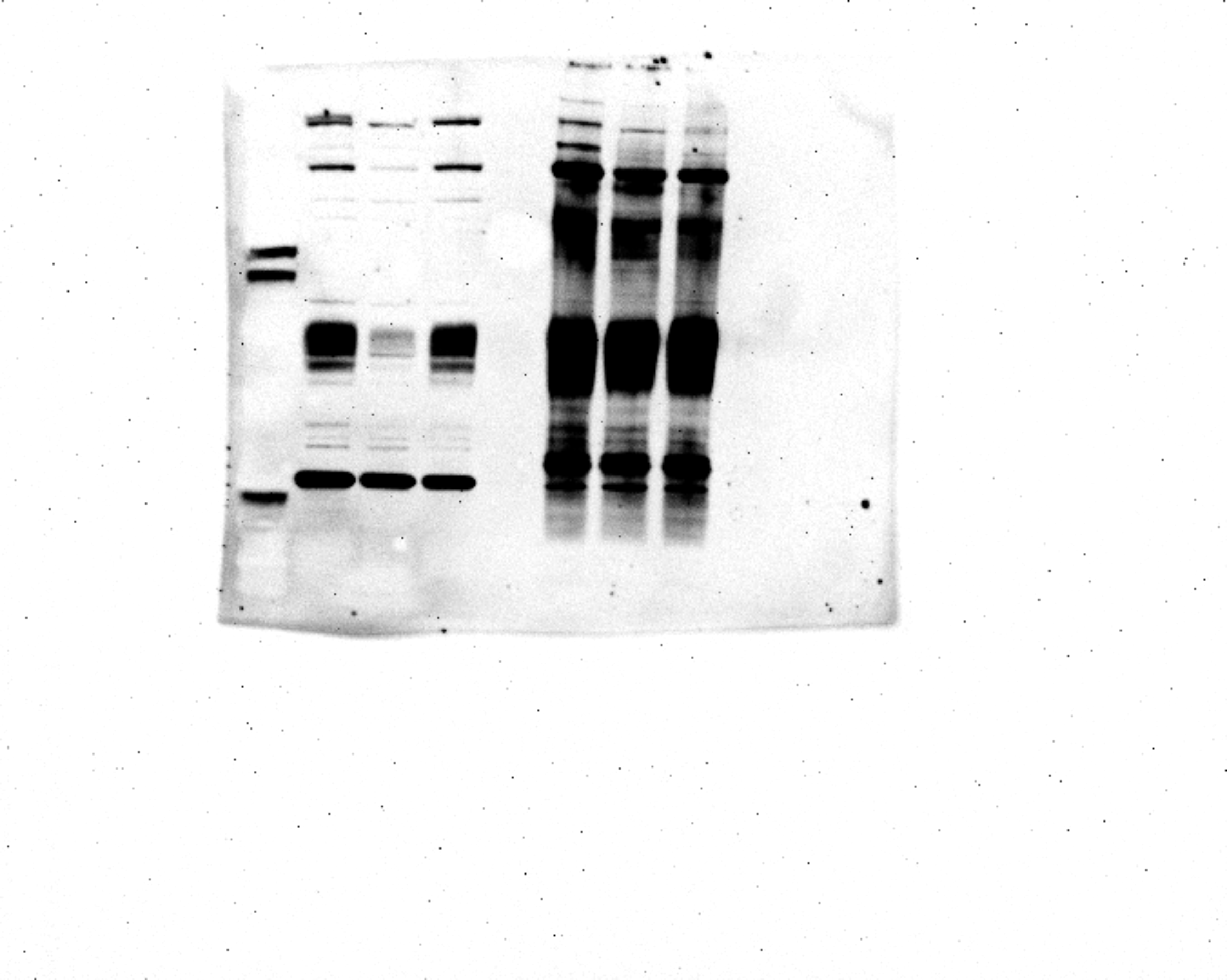

Supplement: Source data 1. [file elife-67828-data1.zip › original Figure 3D.tif]

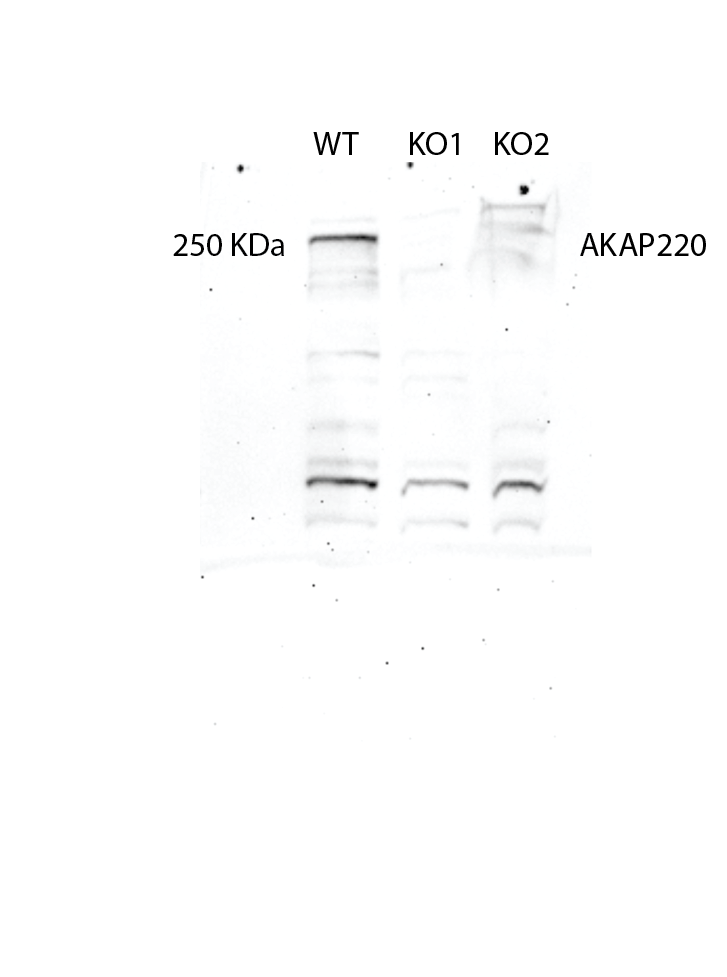

Supplement: Source data 2. [file elife-67828-data2.zip › figure 1K (2).png]

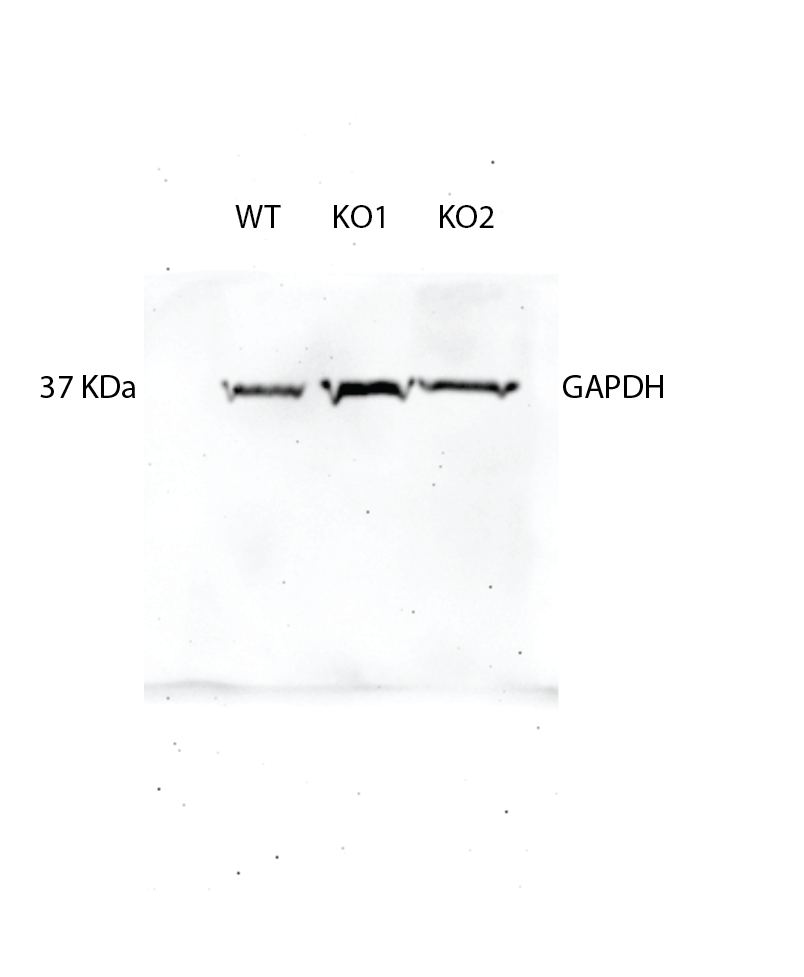

Supplement: Source data 2. [file elife-67828-data2.zip › figure 1K.png]

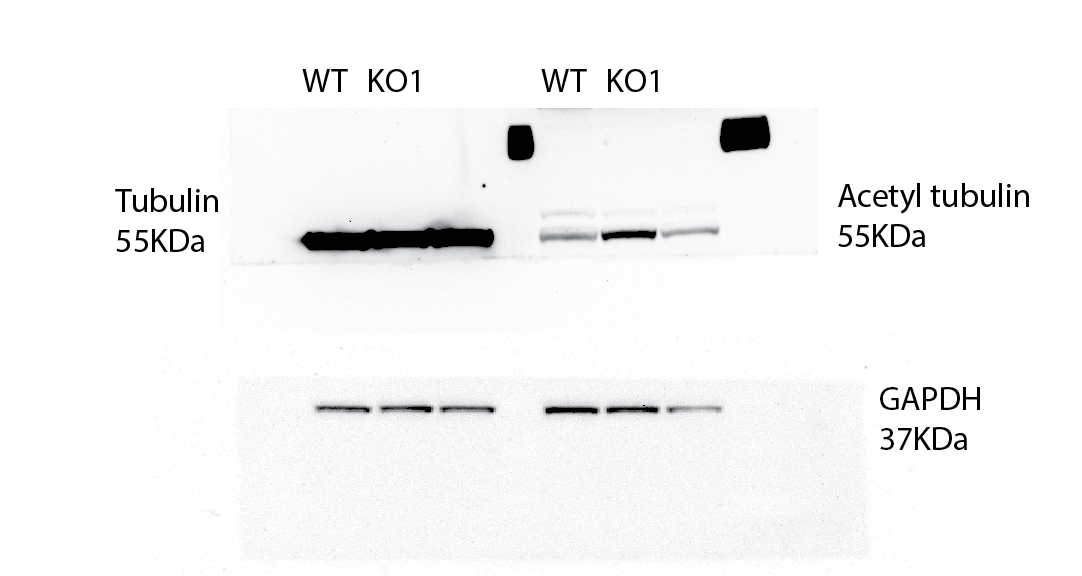

Supplement: Source data 2. [file elife-67828-data2.zip › figure 2C.png]

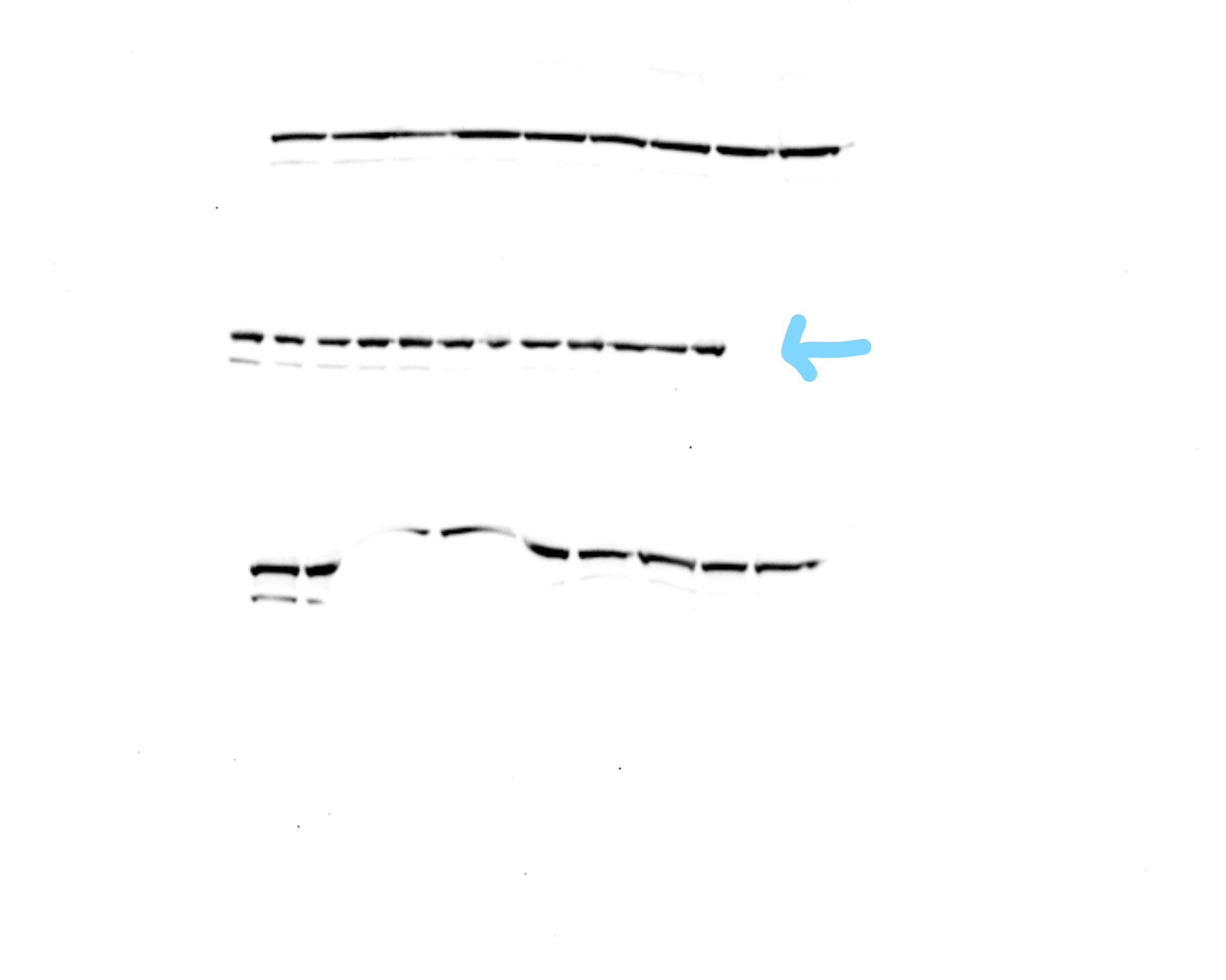

Supplement: Source data 2. [file elife-67828-data2.zip › Figure 2P.jpg]

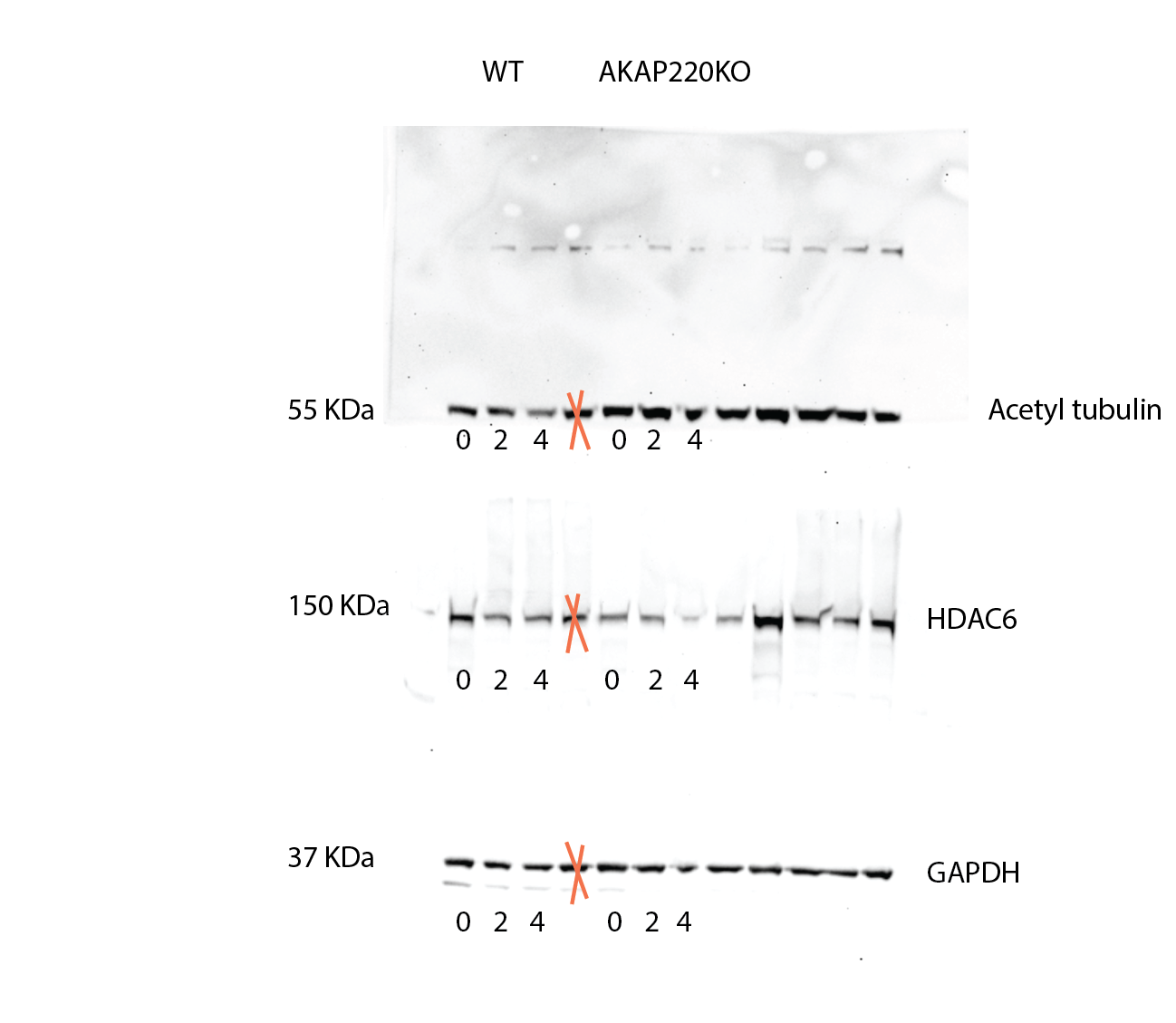

Supplement: Source data 2. [file elife-67828-data2.zip › figure 2P.png]

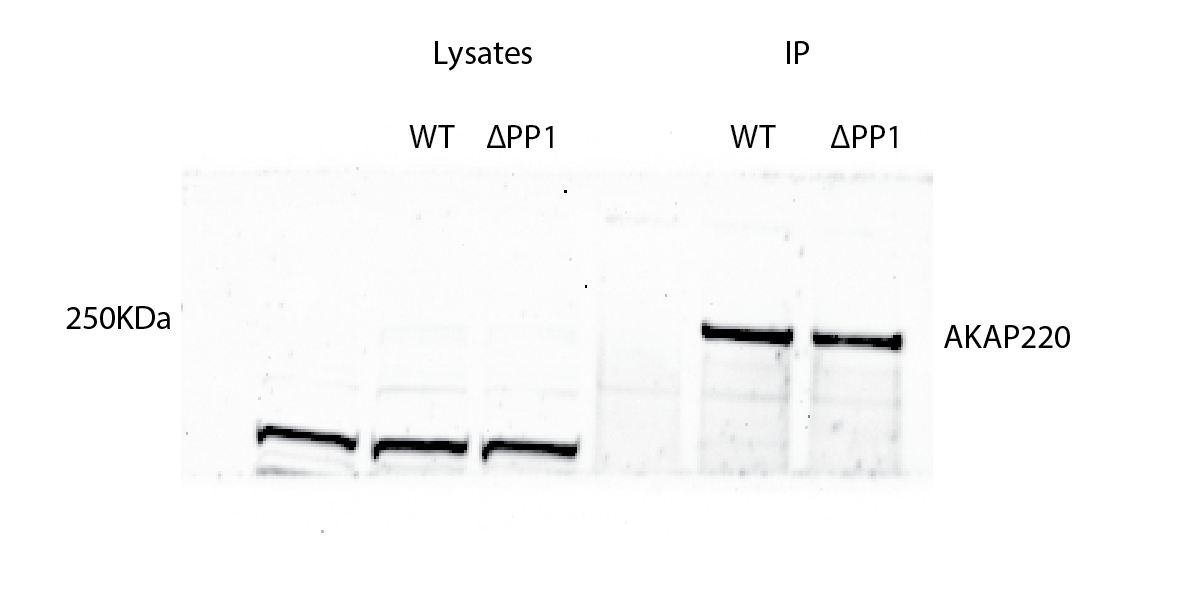

Supplement: Source data 2. [file elife-67828-data2.zip › figure 3- figure supplement 1B.png]

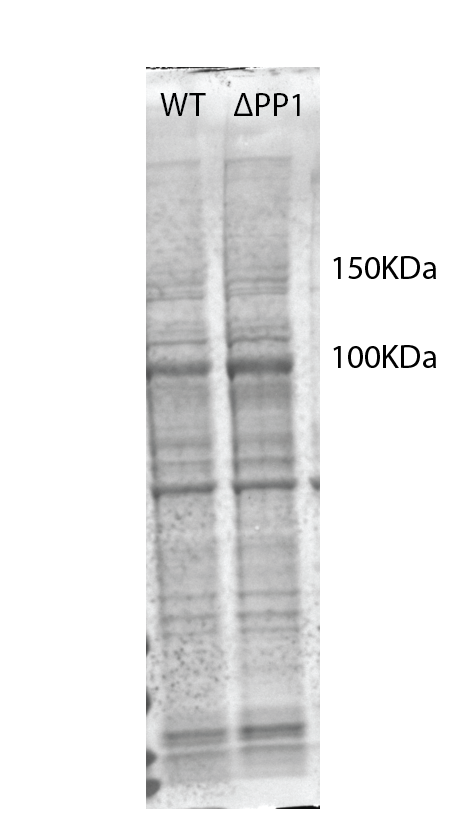

Supplement: Source data 2. [file elife-67828-data2.zip › Figure 3- figure supplement 2A.png]

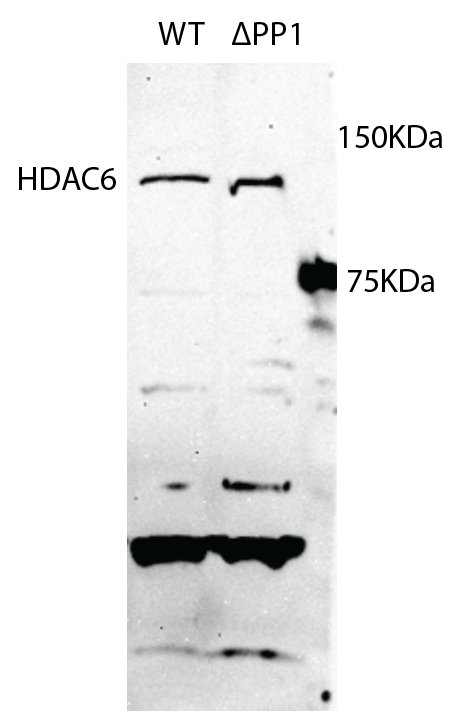

Supplement: Source data 2. [file elife-67828-data2.zip › Figure 3- figure supplement 2B (2).png]

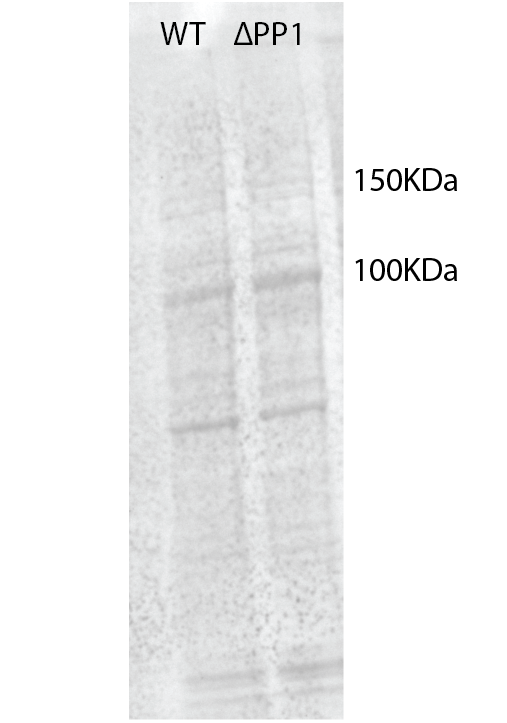

Supplement: Source data 2. [file elife-67828-data2.zip › Figure 3- figure supplement 2B.png]

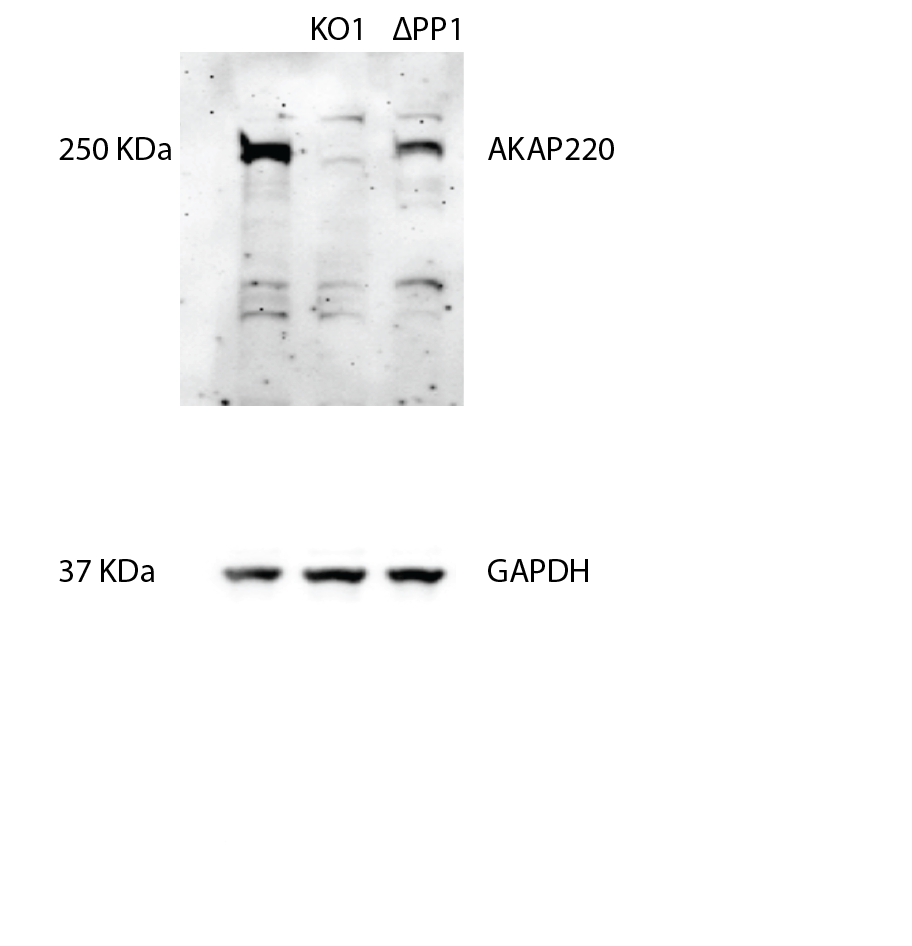

Supplement: Source data 2. [file elife-67828-data2.zip › figure 3C.png]

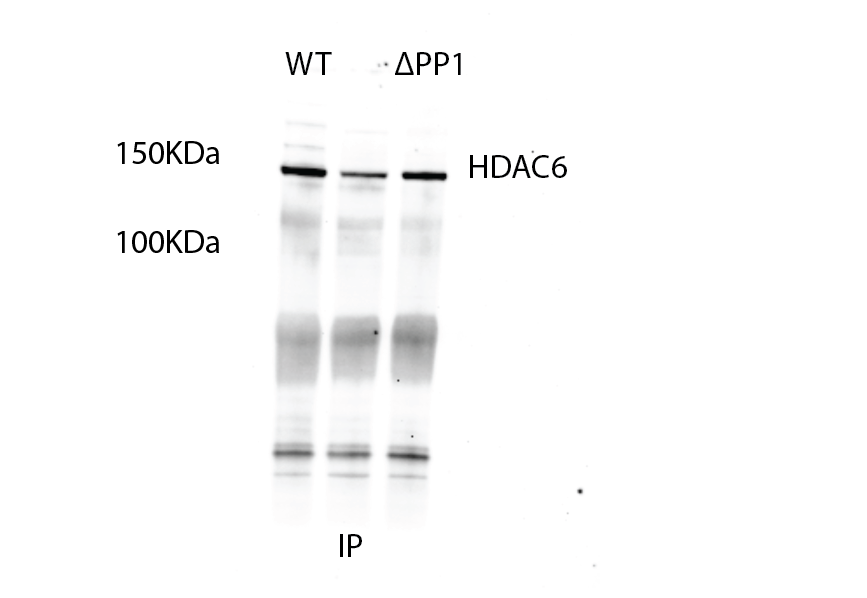

Supplement: Source data 2. [file elife-67828-data2.zip › Figure 3D (2).png]

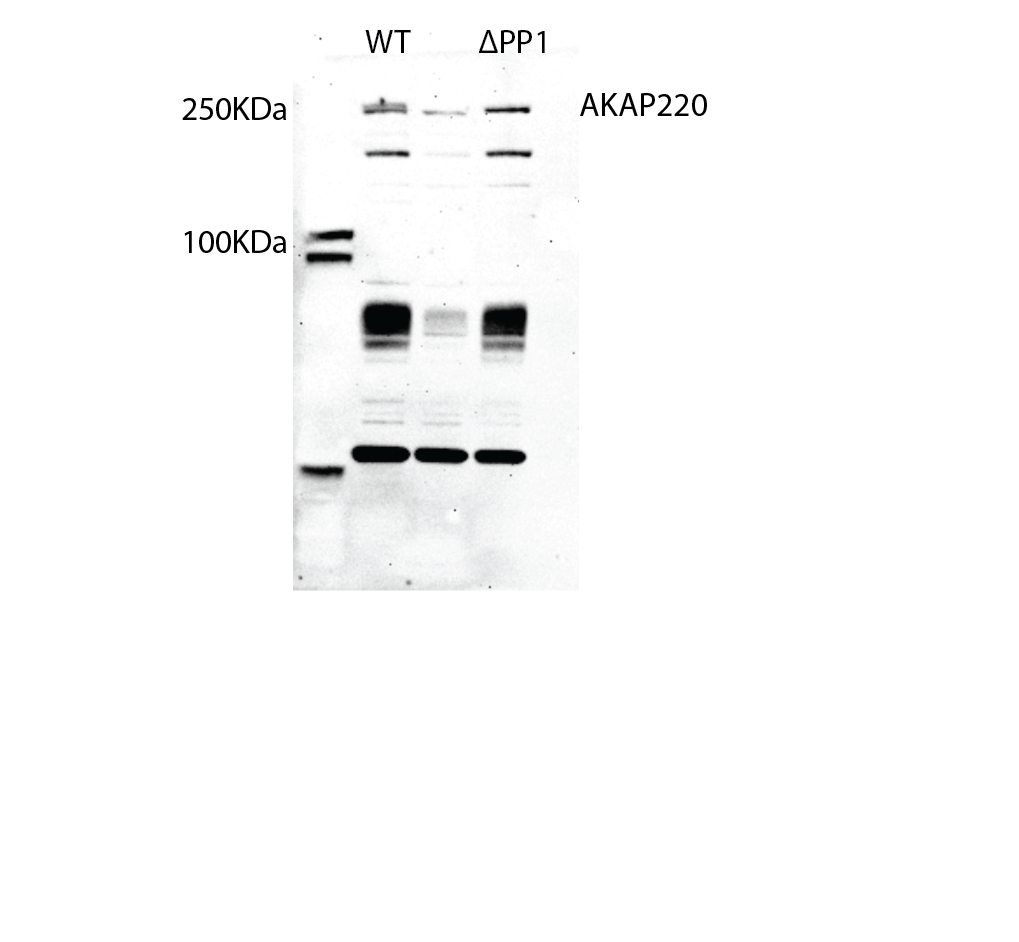

Supplement: Source data 2. [file elife-67828-data2.zip › Figure 3D (3).png]

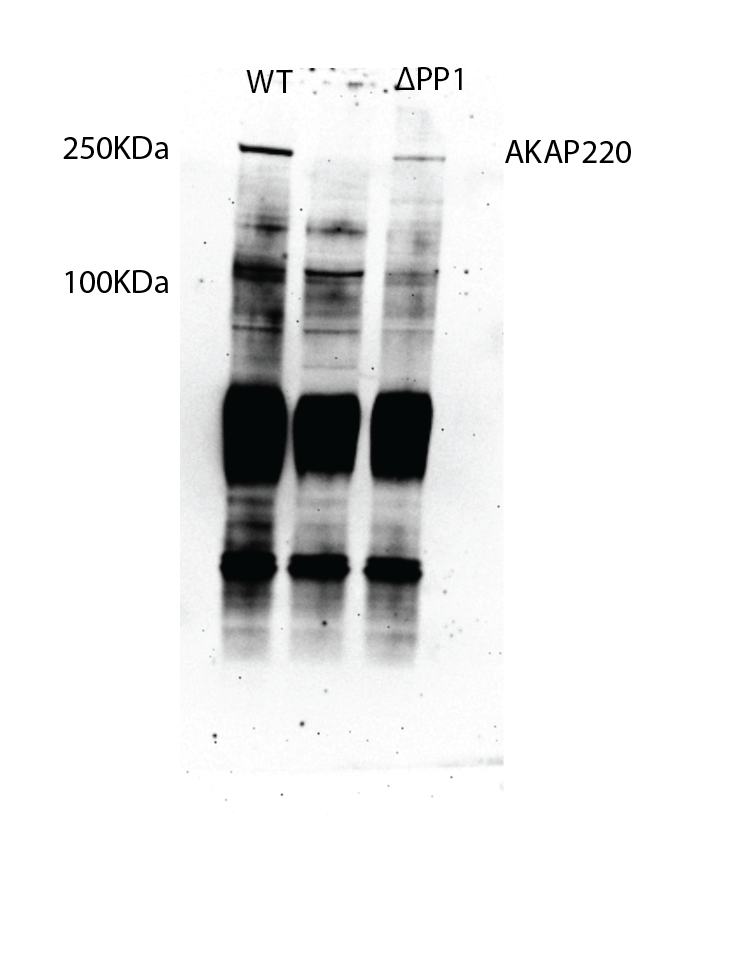

Supplement: Source data 2. [file elife-67828-data2.zip › Figure 3D (4).png]

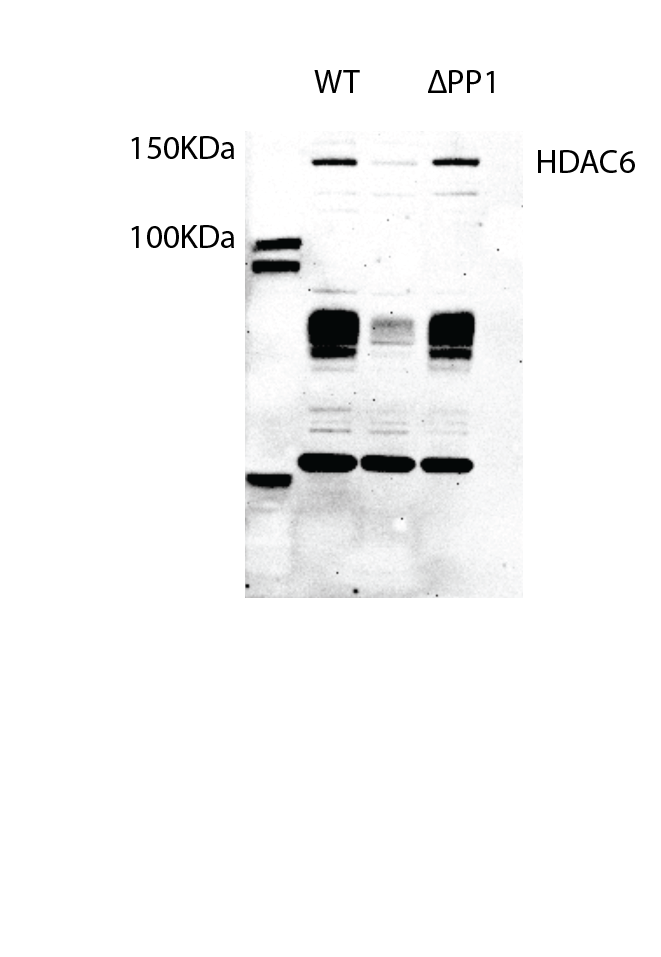

Supplement: Source data 2. [file elife-67828-data2.zip › Figure 3D (5).png]

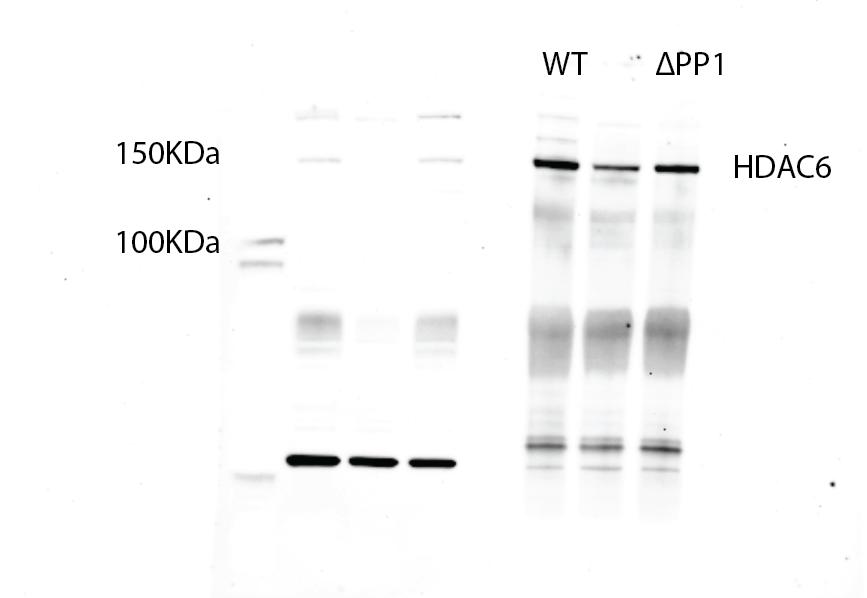

Supplement: Source data 2. [file elife-67828-data2.zip › Figure 3D.png]

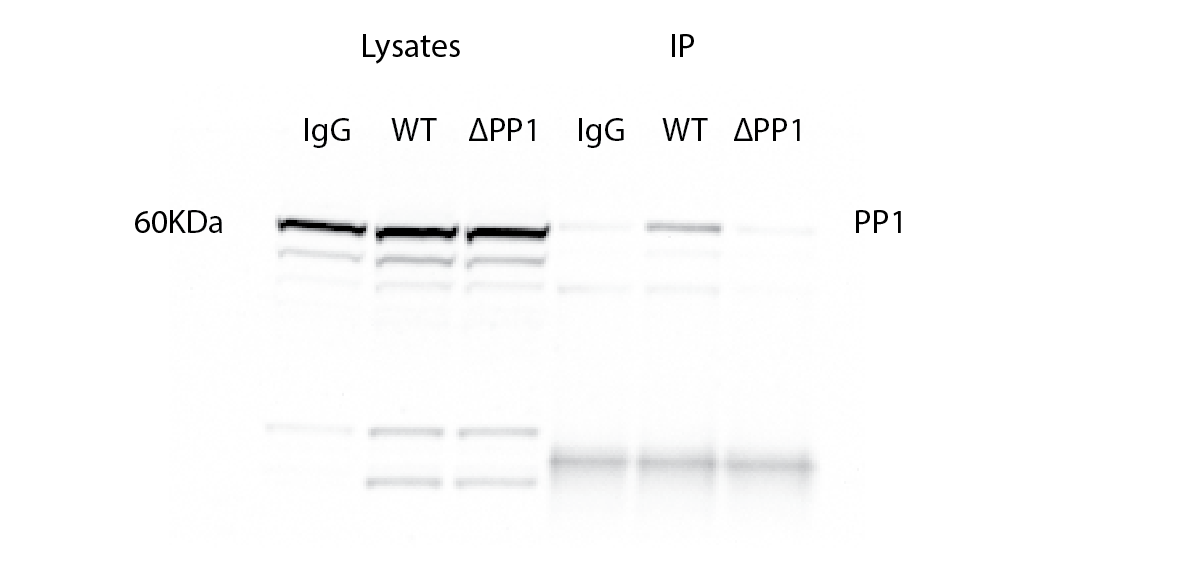

Supplement: Source data 2. [file elife-67828-data2.zip › figure 3-figure supplement 1B.png]

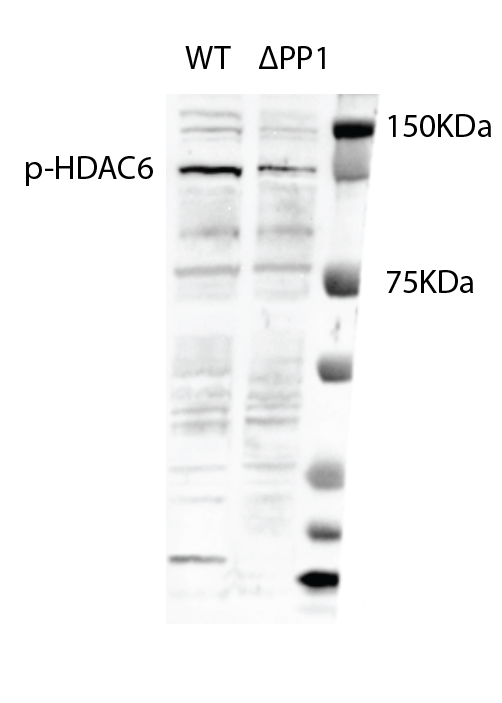

Supplement: Source data 2. [file elife-67828-data2.zip › Figure 3-figure supplement 2A.png]
